# Analysis of Myelin Basic Protein Fragmentation by Proteasome 

**Published:** 2009-04

**Authors:** A. V. Bacheva, A. A. Belogurov, N. A. Ponomarenko, V. D. Knorre, V. M. Govorun, M. V. Serebryakova, A. G. Gabibov

**Affiliations:** 1 Chemistry Department, Moscow State University;; 2 Shemyakin and Ovchinnikov Institute of Bioorganic Chemistry, Russian Academy of Sciences;; 3 Proteomic Center, Russian Academy of Medical Sciences, Institute of Physical–Chemical Medicine

## Abstract

The proteasome is a high molecular protein complex whose purpose is specific protein degradation in eukaryotic cells. One of the proteasome functions is to produce peptides, which will then be presented on the outer cell membrane using main histocompatibility complex (MHC) molecules of the first or second class. There are definite reasons to believe that proteasome directly takes part in the specific degradation of myelin basic protein (MBP), which make up to 30% of all proteins in the myelin sheath of neuronal axons. The details of the proteasomal degradation of MBP are still unclear. In this work, the features of specific MBP degradation by proteasome were studied.

It was demonstrated that MBP (non-ubiquitinated) is a good substrate for 20S and for the 26S proteasome. This is the first work on detecting the sites of MBP proteolysis by proteasome from brains of SJL/J/J and Balb/C mice's lines. Substantial differences in the degradation pattern of this neuroantigen were found, which could indicate the better presentation MBP parts on MHC molecules in the case of mice predisposed to the development of experimental autoimmune encephalomyelitis.

## INTRODUCTION

Multiple sclerosis (MS) – a chronic neurodegenerative disease of autoimmune nature – is an outstanding medical–social problem, because it affects mainly the young and middle-aged. The problem of MS treatment still has no satisfactory solution, and to this day there are several medi-cines (therapies) able to suppress MS to some extent, but not to fully cure it. Neuronal degradation occurs in the brain of MS patients due to the destruction of the neuron's myelin sheath. One biochemical characteristic which differentiates myelin from other biological membranes is the high lipid/protein ratio. Proteins comprise 25–30% of the mass of the myelin sheath dry matter. About 30% of all myelin proteins are three iso-forms of the myelin basic protein (MBP). MBP is one of the main autoantigen in MS. Earlier, we and other authors showed that catalytic anti-bodies [2–5] and some proteases [6–9] may be involved in MBP degradation. It is known that every eukaryotic cell contains a special compart-ment for targeted protein degradation (proteasome), which is a high molecular protease complex. One of the proteasome's functions is to produce peptides, which will then be presented on the cell membrane using main histocompatibility complex (MHC) molecules of the first or second class [10]. There are definite reasons to assume that proteasome directly takes part in specific MBP degradation. The details of this process are still unclear. In this work, the specific features of MBP degradation by proteasome were studied. 

It is well-known that the 20S proteasome (a multicatalytic proteinase complex) is an oligomeric high-molecular-weight (700 kDa) proteinase that can be isolated separately. This complex is the catalytic core of the larger 26S proteasome, which also contains one or two regulatory 19S subunits. It was shown that both 20S and 26S proteasomes are able to degrade proteins, including the MBP [11, 12]. The question of the site-specificity of MBP degradation by the proteasome remained open. It is also known that, during many inflammatory pathological processes, the standard protease com-plex (constitutive proteasome) transforms into a form of immunoproteasome, which has an alternative specificity and catalytic efficiency with re-spect to intracellular proteins processing. It is very likely that this ‘switching' is closely related to different antigen presentation in healthy and in the pathological states. The pattern of MBP degradation by proteasome has not been studied before. 

## RESULTS AND DISCUSSION

**Fig. 1.**
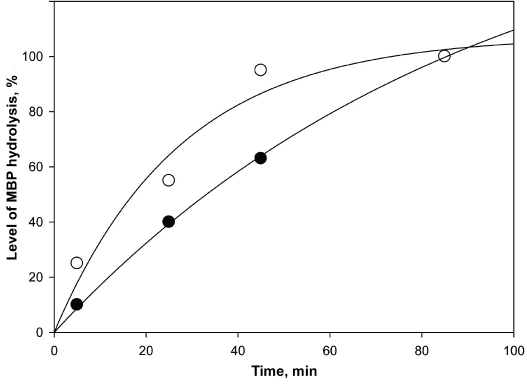
The time dependence of the level of MBP hydrolysis by proteasome. Labels: º 20S proteasome, • 26S proteasome, isolated from outbreed mice liver.

Proteasome was isolated and purified using the technique described in [13] with slight modifications. At the first stage, the degradation of MBP (from bovine brains, isoform with MW 18,5 kDa) was performed by a full 26S complex and catalytic 20S subparticle isolated from mice liver. It is shown in Fig. 1 that the incubation of MBP with 20S and 26S proteasomes leads to progressive MBP degradation. The 20S proteasome completely hydrolyzed MBP in 45 min, while the 26S proteasome requires 85 min to degrade the same amount of MBP. The variation in reaction rates could be ascribed to different proteasome concentrations: in the case of 20S proteasome, the enzyme/substrate ratio was 2.7 : 1 (μg/μg of protein) or 1 : 14.5 (mol/mol); in the case of the 26S proteasome, the enzyme/substrate ratio was lower, namely 1 : 1 (μg/μg of protein) or 1 : 110 (mol/mol). The pro-teasome amount was estimated by the Lowry method, using bovine serum albumin as a standard. 

The MBP hydrolyzates, processed using 20S and 26S protease complexes from the liver of outbreed mice, were fractionated by reverse-phase HPLC on C4 column (Waters, Delta- Pak, 300 Å). Although the general patterns of elution profiles of hydrolyzates were similar, several differences in elution profiles were observed. In particular, it should be noted that some peaks that concurred for 20S and 26S proteasomes differed in their amount of matter; moreover, in the 26S hydrolyzate, new fractions appeared. Thus, the 26S degradation pattern of MBP somewhat altered compared to the 20S pattern. These differences can be explained by the unequal accessibility to proteolysis of MBP sites located on the surface and in the depth of the protein globule, as well as by their pronounced secondary structure. In the case of the 26S proteasome, the accessibility of different sites of MBP was not of great importance, because the 19S subparticle contains subunits responsible for the denaturation of protein molecules that are to be degraded. 

**Fig. 2.**
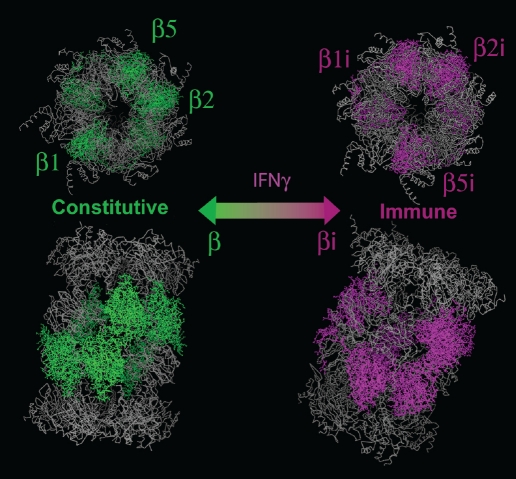
The proteasome–immunoproteasome equilibrium. Immune catalytic subunits produced in cells exposed to interferon gamma.

The pool of proteasomes is heterogenous and consists of macromolecular complexes of several types, with catalytic subunits referred to the so-called constitutive type (β1, β2, and β5) or immune type (β1i, β2i, and β5i) (Fig. 2). Six catalytic subunits of proteasome expressed three types of activities, namely chymotrypsin-like (cleavage of the peptide bond on the carboxyl site of hydrophobic and aromatic amino acids Leu, Tyr, Phe), trypsin-like (hydrolysis after positively charged Lys and Arg), and caspase-like (hydrolysis after negatively charged Asp and Glu) [14]. 

The constitutive-to-immunoproteasome ratios have clearly defined tissue-specificity and, to a considerable degree, depend on the immune state of the organism. For example, more than 90% of the total amount of proteasomes in the brain are constitutive, but in the spleen about 90–95% of pro-teasome are immunoproteasomes. Besides, in any tissue under interferon gamma exposure, the immune catalytic subunits are produced extensively, being integrated into the newly assembled multicatalytic complexes [15]. 

It was shown earlier that replacing constitutive catalytic subunits with immune leads to a change in hydrolysis specificity and to an increase in its velocity. Immunoproteasome complexes almost lose their ability to hydrolyze peptide bonds after aspartic and glutamic acids residues (caspase-like activity);however, a lot more often the hydrolysis takes place after hydrophobic and, especially, branched hydrophobic amino acid residues. There-fore, during immunoproteasome processing, an increased amount of peptides bearing hydrophobic amino acids on the C-end is produced. Because hydrophobic amino acids on the C-end of peptides are important anchor fragments for binding with MHC I class molecules, the change in hydrolysis specificity leads to an increase in the production of peptides able to form complexes with MHC molecules. Then, the fragments bounded to MHC are presented on the outer cellular membrane to immune cells. Thus, cells bearing immunoproteasome should more effectively present their own anti-gens. 

It is also known that α-subunits of the proteasome catalytic 20S subparticle function as a gate forming an axial channel that regulates the influx and efflux of proteins and their degradation products by the opening and closing of the entrance to the so-called catalytic chamber. Therefore, the closing of this channel may enable complete substrates degradation by preventing the efflux of partially hydrolyzed peptides [16]. It was also shown [17] that the opening of the channel strongly affects the proteolysis kinetics and the distribution of hydrolytic fragments obtained in vitro with re-spect to the peptide length. If the channel is open, the hydrolysis rate increases; however, in this case, the mean length of produced fragments also increases by 40%. Thus, the higher hydrolysis rate of immunoproteasome should result in longer degradation products (peptides), which will better bind to MHC molecules and, thus, will more effectively be presented on the cell's surface. 

It is well established that SJL/J mice are genetically predisposed to the development of experimental autoimmune encephalomyelitis. This pathology is the clinically relevant animal model of multiple sclerosis (MS). Using immunoblotting we studied the composition of the protea-some pool in the brains of SJL/J mice, and it was revealed that the immunoproteasome amount is increased in comparison to Balb/C mice brains (data not shown). Therefore, at the next stage of the study, the proteasomes from the brains of SJL/J and Balb/C mice were isolated, and the proteolysis of MBP by those proteasome samples was studied. 

**Fig. 3.**
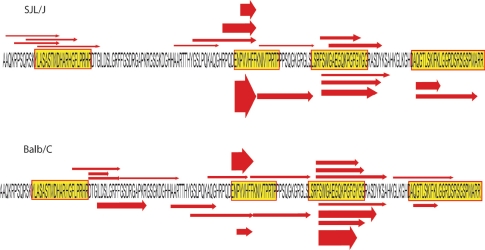
Amino acid sequence of MBP. Arrows mark the proteolytic peptides determined by chromato-mass spectrometry in hydrolyzates of MBP by proteasome from the brains of SJL/J/J mice (upper picture) and of Balb/C mice (lower picture). The arrow's thickness indicates the frequency of occurrence of the corresponding peptide. Color rectangles show the immunodominant regions of MBP.

The MBP hydrolyzates obtained using proteasomes isolated from mice of the two strains were studied by LC-MS. Figure 3 shows the complete amino acid sequence of MBP, and the arrows indicate the major fragments produced by proteasome pools from the above-mentioned sources. The thickness of the arrows indicates the relative amount of corresponding peptide in hydrolyzate. 

In the MBP sequence, several regions could be attributed to immunodominant protein regions: 12-31, 82-98, 110-128 and 144-169; fragment 85-98 is the so-called encephalitogenic epitope, which can activate the immune response. 

It should be noted that MBP degradation by proteasome from outbreed mice liver, both by the full 26S complex and the catalytic core, does not lead to the generation of immunogenic epitopes, all of which were exposed to subsequent fragmentation inside the proteasome catalytic chamber. 

In MBP hydrolyzate by proteasome isolated from the brains of SJL/J and Balb/C mice, almost the only hydrolytic site where proteasome exhibits its caspase-like activity was the bond between amino acid residues Asp81-Glu82, close to the beginning of the encephalitogenic peptide. 

The MBP hydrolysis pattern by proteasome isolated from the brains of SJL/J and Balb/C mice differed to some extent. In the brains of the auto-immune mice, the generated epitopes could be considerably better colocalized with immunodominant regions of the protein. Under the action of the SJL/J mice proteasome pool on the myelin basic protein, up to a quarter of all obtained hydrolytic fragments were made up of encephalitogenic pep-tides. The Balb/C mice strain proteasome produces half the amount of that peptide. Moreover, fragments obtained using a Balb/C mice-brain protea-some poorly correlate to the recognition regions of MHC class II molecules. 

**Fig. 4.**
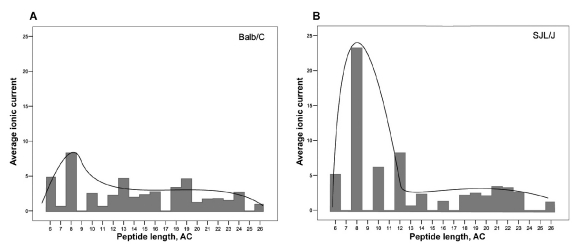
The length distribution of peptides found in MBP hydrolyzates obtained using proteasome pools from the brains of (a) Balb/C mice and (b) SJL/J/J mice. Bars on the diagram correspond to the experimental value of the ionic current for the peptides of relevant lengths as determined by LC-MS.

Figure 4 shows the length distribution of peptides in corresponding hydrolyzates. On the vertical axis, the experimental values of the ionic current obtained using LC-MS for a peptide of a given length is shown. It can be seen that the maximum of the distribution is on peptides having 8 amino acids in length, both in case of proteasome isolated from the brains of SJL/J or Balb/C mice. However the relative values of the average ionic current for these two samples differed drastically, which can be an argument for substantially more fragments of the given length in the case of autoimmune mice. In the hydrolyzates studied, peptides with an even number of amino acid residues predominate, and no peptides shorter than 4 residues were detected. These data are in good agreement with the literature [18] and with the opinion that one of the main roles for proteasome in cells is the gen-eration of peptides for subsequent presentation on MHC class I molecules, which can bind peptides of 8–10 amino acids in length. Longer peptides found in hydrolyzates could be further processed to shorter fragments inside the cell and presented on the MHC I class molecules, and they could also participate in presentation on MHC II class molecules [10]. 

## CONCLUSIONS

In conclusion, in our work it was shown that both 20S and 26S proteasomes are able to hydrolyze the myelin basic protein, and the proteasome/MBP molar ratio was found to be 1 : 14.5 and 1 : 110, respectively; the complete hydrolysis time was 45 and 85 min, respectively. After separating hydro-lyzates by HPLC, the molecular weights of the fragments were determined by MALDI mass spectrometry. After analyzing the amino acid sequence of MBP, the proteolytic sites were identified. It was demonstrated that the nonubiquitinated myelin basic protein is a good substrate for both 20S and 26S proteasomes. This was the first work to identify the sites of MBP proteolysis using a proteasome isolated from the brains of SJL/J and Balb/C mice and to show significant differences in the degradation pattern of this autoantigen. These findings could argue for a better presentation of MBP fragments on the MHC molecules in the case of mice genetically predisposed to the development of experimental autoimmune encephalomyelitis. 

## Acknowledgements

This work was supported by RFBR grant 07-04-12100-ofi, 09-04-01546-a, 07-04-92168-NCNI_а, NATO SFPP 982833, and the "Fundamental science for medicine – 2008" program of the presidium of the Russian Academy of Sciences.
